# Kinematic Comparisons of Increased Exercise Repetitions and Intensities on the Dominant and Non-Dominant Upper Limbs for Prevention of Dyskinesia

**DOI:** 10.18502/ijph.v49i10.4690

**Published:** 2020-10

**Authors:** Haemi JEE

**Affiliations:** Department of Physical Therapy, Namseoul University, Cheonan-si, Korea

**Keywords:** Repetition, Exercise intensity, Asymmetry, Dyskinesia

## Abstract

**Background:**

Increased exercise repetitions and intensities need to be compared between dominant and non-dominant sides to prevent asymmetrically conducted movements for possible dyskinesia.

**Methods:**

A total of 20 participants were enrolled from Inha University, Incheon, Korea in 2019. They were assessed for comparisons of asymmetrical motion between the dominant and non-dominant arms during the abduction and adduction lateral raises during more than fifteen repetitions and low and high exercise intensity by giving different weight loads based on 1-RM.

**Results:**

Repetition led to significant reductions in range of motion for both dominant and non-dominant sides. In addition, increased repetitions led to significant greater reductions in range of motion especially toward the last phases of repetitions. Moreover, the dominant side showed significantly increased accelerations with increased intensities.

**Conclusion:**

Increased repetitions and exercise intensity led to reduced range of motion and increased accelerations especially for the dominant sides. Dispersing kinematics should be considered to minimize possible dyskinesia between the symmetric sides when performing repetitive and loading physical activity.

## Introduction

Dyskinesia may come from various causes including improper exercise habit. Such dyskinesia is more prevalent in athletes who predominantly use upper limbs (1). Repeated usage of the dominant side with greater weight load promote comparatively greater usage and development of the musculatures of the dominant side in comparison to the non-dominant side. Such imbalance in usage and development may lead to asymmetry of the movement ability and posture between the sides. Evaluation of asymmetry in movement and posture can detect abnormal progression in muscular function during the early phase prior to the advancement into musculoskeletal pathology (2–5).

Studies of comparisons between dominant and non-dominant side have previously been conducted (5–8). Repeated motions leading to abnormality between ipsilateral and contralateral sides in athletes have been reported in various sports that require greater usage of one side. Such excessive or erroneous usage of one side have also been reported to lead to dysfunctional asymmetry (6, 7, 9).

Although various studies have been conducted to observe the effects of greater usage of the dominant side on the movement ability and function, it seems that no study extended the study design to observe the effects of increased repetitions and intensity. That is, exercise ability can be altered by changing exercise components such as frequency, intensity, time, and type (FITT) (10). Increased repetitions or intensity during exercise have been known to promote different physiological responses. Moreover, excessively increased fatigue during exercise may promote disturbance of postural control and muscular (11). Previous studies rarely considered these FITT principle components for progression of asymmetry in movement imbalance between the dominant and non-dominant sides (12). The exercise components have been utilized by exercise specialists to provide exercise intervention for altering bodily musculatures and related physiological components.

Shoulder joint motions were targeted in this study since the shoulder girdle with the upper extremities have commonly being exposed to asymmetric dyskinesia (5, 13). Identical movements by the dominant and non-dominant upper extremities were performed and compared with increased repetitions and intensity to observe kinematic changes during abduction and adduction motions. Initial positions during the abduction and adduction motions were observed by the instantaneous angle (°) and alterations in motions were observed by the angular acceleration (deg/s^2^) to observe the effects of fatigue on exercise-induced kinematic changes and corresponding dyskinesia

## Materials and Methods

### Subjects

The study was approved by the Inha University Ethics Committee and performed according to the Declaration of Helsinki. All participants were fully and thoroughly informed of the purpose and procedure of the experiment prior to the assessments. Verbal and written informed consents were obtained from the participants to participate in the study.

Twenty right-handed participants without clinically significant shoulder pain or dysfunction were first recruited from Inha University, Incheon, Korea in 2019. They aged between 19 to 28 yr with mean age of 22.5 (±2.33) yr and mean body mass index (BMI) of 21.7 (±2.29) kg/m^2^. They participated in performing lateral raise with two dumbbells held on both hands. The participants performed in physical activity 2 to 3 d per week with the average exercise time of 30 min.

### Experimental procedure

The participants were asked to restrain from heavy physical activity and consume small-sized meal prior to the assessment. Procedure for repeated lateral raises was explained and shown to the participants for proper motion and timing of movements (14). Borg scale (0 to 10) was used to measure the rate of perceived exertion (RPE) to measure the exercise intensities (14, 15).

### Questionnaires

Anthropometric measures such as weight (kg) and height (g) were obtained. General information such as age (age), health status, and exercise habit were also obtained through prepared questionnaires. Laterality quotient for dominant side was assessed with the Edinburgh Inventory and the participants with scores more than 6 or above were included (16, 17).

### Lateral raises

Two types of dumbbell weights were selected for low (≤40% of 1-RM (repetition maximum)) and high (70% of 1-RM (repetition maximum)) intensity exercise for strength and hypertrophy (18). One repetition maximum based exercise intensity was selected from previously reported 1-RM calculation method (19). The participants were asked to try to dumbbells weight ranging from one to six kilograms. All lateral raise motions were shown for slow and controlled motions with explanation for safety and proper motion. The lateral raises were initiated with erect position with the arms straight and slightly apart with each dumbbell held by each hand and feet shoulder-wide apart (8). The arms were abducted at the same time until slightly above the horizontal shoulder level with the range of motion between 10 to 100 degrees without a jerking motion. A set of abduction and adduction motion was repeated at least fifteen times or until failure due to fatigue. In addition, if the lateral raise not fully performed or abduction motion was less than 90 degree, the motion was not counted and consider termination of the set (15).

### Motion assessment

A set of two synchronize devices (CC2650 SensorTag from Texas Instruments) with excellent reliability (r<0.9) were attached to the distal radioulnar joints of the wrist prior to the lateral raises (20–22). Angles (°) and angular accelerations (deg/s^2^) were assessed and recorded to a nearby computer program real-time via Bluetooth every one-tenth of a second. Out of the collected data, initial abduction and adduction data were obtained and compared between the low and high intensity trials and between repeated every three to four trials.

### Statistical Analysis

The study sample size was determined based on previous studies that assessed lateral raises of the upper limbs (5, 8). Ten or more subjects were used for the assessments (5, 8). The normality analysis was performed using the Shapiro-Wilk test prior to the comparative assessment. After observing normal distribution of the data, oneway ANOVA (analysis of variance) was performed to compare between the dominant and non-dominant arms and between the initial repetition, every two to three repetitions, and final repetitions for a total of five repetitions for the effects of fatigue on the repeated performance. The assessment results were given as means ± standard deviations (SD). Statistical significance was accepted for the *P*-value of 0.05.

## Results

**Fig. 1:**
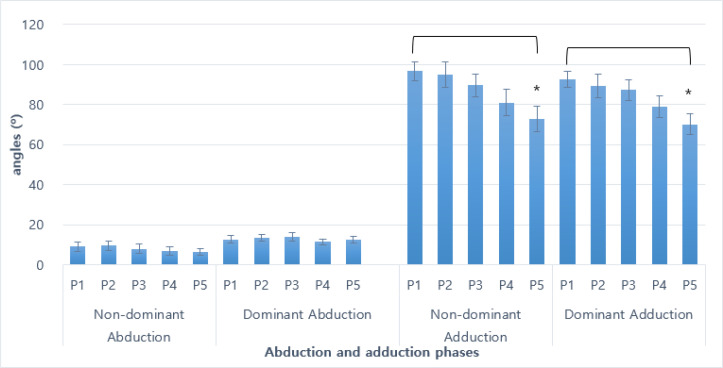
Changes in initial abduction and adduction angles during repetitive lateral raises ^*^: *P*<0.05, P1–P5: phase 1 through phase 5 (first to every 3 to 4 abductions or adductions), non-dominant: left arm, dominant: right arm

Fifteen or more repeated lateral raises composed of abduction and adduction shoulder motions were compared between the dominant and non-dominant arms and repetitions. First, the overall angle or positional comparisons between left and right and repetitions are shown in Fig. 1.

During the abduction phases, significant differences in angle were shown during the last repetitions of the adduction motions. Last phase of the repetitive adductions shown significant reduction in angles in comparison to the initial phase in both the dominant (96.8 (±4.72) vs. 72.9 (±6.62) degrees; *P*<0.050) and non-dominant sides (92.6 (±4.20) vs. 70.1 (±5.25); *P*<0.029).

**Fig. 2:**
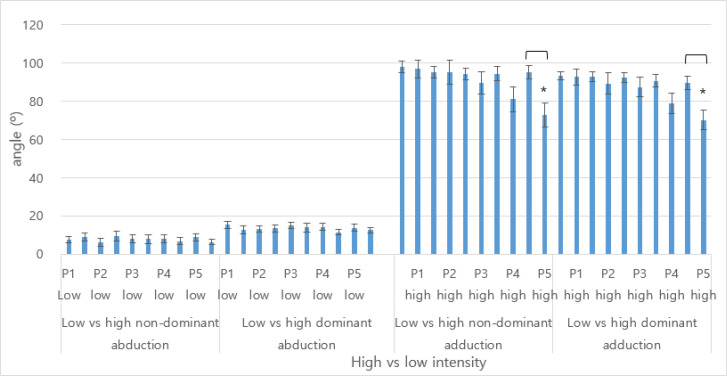
Differences in abduction and adduction angles (°) during low and high load exercise intensities ^*^: *P*<0.05, P1–P5: phase 1 through phase 5 (first to every 3 to 4 abductions or adductions), non-dominant: left arm, dominant: right arm, low: low-load intensity, high: high-load intensity

Figure 2 compared angle during 5 phases of the repetitions. As shown, the low exercise intensity showed consistently showed similar minimum and maximum angles throughout the repetitive phases. However, the high exercise intensity showed rapid decline of the adduction angle toward the last phrase leading to significantly small angles: dominant side (95.2 (±3.38) vs. 72.9 (±6.32) degrees; *P*<0.004) and non-dominant side (89.5 (±3.58) vs. 70.1 (±5.25); *P*<0.005).

**Fig. 3:**
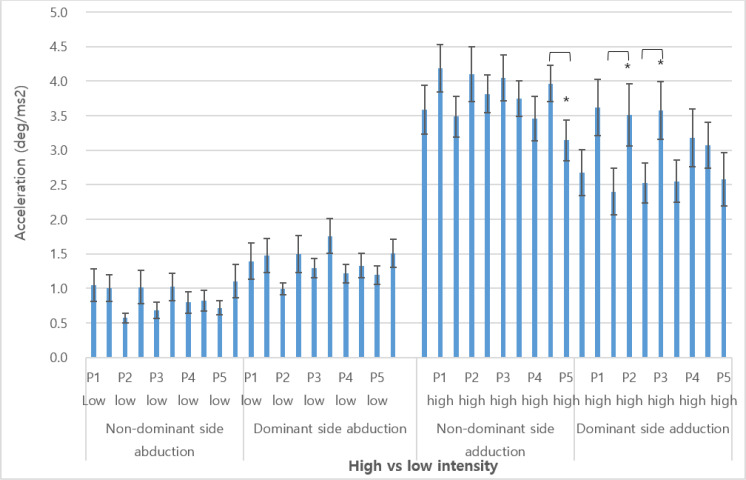
Differences in abduction and adduction accelerations during low and high exercise intensities ^*^: *P*<0.05, P1–P5: phase 1 through phase 5 (first to every 3 to 4 abductions or adductions), non-dominant: left arm, dominant: right arm, low: low-load intensity (40%≥1RM), high: high-load intensity(70%≤1RM)

Figure 3 compares the changes in acceleration between of the initial abduction and adduction phases during low and high intensities. Overall, the high exercise intensities showed faster accelerations in comparison to the low exercise intensities. In addition, significantly slower accelerations were observed during the last phase (3.97(±0.36) vs. 3.15(±0.30) m/ms^2^; *P*<0.046) of the abduction and significantly faster accelerations were shown during the second (2.40(±0.34) vs. 3.51(±0.34) m/ms^2^; *P*<0.050) and third (2.53(±0.29) vs. 3.58(±0.42) m/ms^2^; *P*<0.049).

## Discussion

Improper or imbalance in physical activity or movement have been known to lead to asymmetry between two equal sides of the body and postural imbalance (3, 4, 12, 23). Understanding the kinematics of the equal sides may allow prevention of dyskinesia. This study observed differences in kinematics of two symmetric sides composed of the dominant and non-dominant sides by comparing repetitions that promote fatigue.

Minimum of fifteen lateral raises were continuously conducted by the participants until maximum of twenty lateral raises were performance or discontinued by fatigue. Low and high exercise intensities were measured by utilizing dumbbells with different weights.

First, abductions of all bouts with low and high exercise intensities were compared without significance for the five different phases or repetitions from initial to every three repetitions until the last or fifth repetition. However, significantly smaller angles were observed from the last fifth phase of the adduction motions in comparison to the first phase. In addition, the adduction angles continuously decreased through increased repetitions. Postural changes have been observed previous studies with repetitions due to muscle fatigue and alteration to sensory input (11, 24, 25).

Next, initial abduction and adduction angles were compared between the low and high exercise intensities during the five phases of repetitions. Decline in angles were prominently shown for during the five phases of high intensity abduction and adduction motions. Changes in the initial abduction and adduction angles were rather uniform for the low intensity abduction and adduction motions. However, the angles noticeably decrease as much as 20 degrees for the high intensity abduction adduction motions. Exhaustive local exercise has been known to affect strength loss of more than 25 to 40 percent (11). Disturbed strength, neglect, and/or compensate for muscle fatigue and related postural disturbance (11).

Finally, instantaneous accelerations were compared between the low and high exercise intensities throughout the five phases of repetitions. Overall, accelerations were greater during the high load intensity lateral raises. Significant differences in acceleration were shown during the adduction motions for both the dominant and non-dominant sides. Moreover, the dominant side showed overall significant greater accelerations during majority of the phases. That is, the differences between the high and low exercise intensities were the greatest between dominant sides.

Dominant to non-dominant sides were compared in previous study (5). Previous study showed better control by the dominant side for the range of motion with greater accuracy (5). Strength training that involves high weight loads lead to increased strength, hypertrophy, and power. Various structural and neural factors. Exhaustive exercise disturbances at the level of muscle activation that includes neuromuscular junction, excitation, metabolite accumulation, glycogen depletion, and action potential propagation (11). Disturbances in the neuromuscular system lead to changes in the range of motion and movement velocity. Fatigue in the local muscle lead to alteration of the proprioception and deterioration of muscle contractibility due to degradation of neuromuscular control (11, 26, 27). In order to compensate degraded neuromuscular control, body compensate postural changes by adjusting or redistribution of activate muscle groups (27). Furthermore, previous reports on dominant sides delineate more torque-efficient movements for better accuracy and dynamic control in comparison to the non-dominant sides due to greater entropy in everyday situations (12, 28).

However, as the repetitions increase, the dominant side showed greater changes in range of motion. This could be explained by the speed-accuracy trade-off, resulting in increased speed with reduced accuracy (5, 23). Although the dominant sides have more strength and motor control ability in comparison to the non-dominant sides, the dominant sides may fluctuate speed to sacrificing speed for enhanced accuracy in motion (23, 29).

Various studies on athletes participating repetitive one sided motion reported asymmetry or imbalance at young age (3, 4, 12, 20, 23).

## Conclusion

Increased exercise intensities and repetitions may lead to greater reduction in accuracy in range of motion and reduction in accelerations. Such results indicate need for considerable care in weight load for exercise intensity for both the dominant and non-dominant sides. Changes in acceleration and range in motion should be monitored for asynchronized movements for prevention of exercise related dyskinesia. Therefore, early detection for promoting muscular imbalance and asymmetry in motion should be carefully observed and intervened for proper compensatory action.

## Ethical considerations

Ethical issues (Including plagiarism, informed consent, misconduct, data fabrication and/or falsification, double publication and/or submission, redundancy, etc.) have been completely observed by the authors.
